# Examiner and simulated patient ratings of empathy in medical student final year clinical examination: are they useful?

**DOI:** 10.1186/1472-6920-14-199

**Published:** 2014-09-22

**Authors:** Barry Wright, Jean McKendree, Lewys Morgan, Victoria L Allgar, Andrew Brown

**Affiliations:** Hull York Medical School, John Hughlings Jackson Building, Heslington, York, YO10 5DD UK; Child Adolescent Mental Health Service, 31 Shipton Lane, York, YO30 5RE UK

**Keywords:** Empathy, Medical students, Simulated patients, Final clinical examination, OSLER, OSCE

## Abstract

**Background:**

Many medical schools state that empathy is important and have curricular learning outcomes covering its teaching. It is thought to be useful in team-working, good bedside manner, patient perspective taking, and improved patient care. Given this, one might expect it to be measured in assessment processes. Despite this, there is relatively little literature exploring how measures of empathy in final clinical examinations in medical school map onto other examination scores. Little is known about simulated patient (actors) rating of empathy in examinations in terms of inter-rater reliability compared with clinical assessors or correlation with overall examination results.

**Methods:**

Examiners in final year clinical assessments in one UK medical school rated 133 students on five constructs in Objective Structured Long Examination Record (OSLER) with real patients: gathering information, physical examination, problem solving, managing the diagnosis, and relationship with the patient. Scores were based on a standardized well-established penalty point system. In separate Objective Structured Clinical Examination (OSCE) stations, different examiners used the same penalty point system to score performance in both interactional and procedural stations. In the four interaction-based OSCE stations, examiners and simulated patient actors also independently rated empathy of the students.

**Results:**

The OSLER score, based on penalty points, had a correlation of −0.38 with independent ratings of empathy from the interactional OSCE stations. The intra-class correlation (a measure of inter-rater reliability) between the observing clinical tutor and ratings from simulated patients was 0.645 with very similar means. There was a significant difference between the empathy scores of the 94 students passing the first part of the sequential examination, based on combined OSCE and OSLER scores (which did not include the empathy scores), and 39 students with sufficient penalty points to trigger attendance for the second part (Cohen’s d = 0.81).

**Conclusions:**

These findings suggest that empathy ratings are related to clinical performance as measured by independent examiners. Simulated patient actors are able to give clinically meaningful assessment scores. This gives preliminary evidence that such empathy ratings could be useful for formative learning, and bolsters the call for more research to test whether they are robust enough to be used summatively.

## Background

The medical student admissions process frequently involves attempts at the assessment of empathy [1, 2]. Stepien and Baernstein [3] describe the importance of different elements of empathy. They suggest that in clinical careers there is a need for more than cognitive empathy, defined as the intellectual understanding of another’s perspectives, because high levels of clinical competence also require affective empathy, an emotional understanding and engagement with the patient. Some see the cognitive deployment of empathy in a detached way in medical settings [4] as a key skill and others believe that emotional resonance and affective displays are important [5]. Empathy is widely described as being an essential part of clinical competence representing one aspect of broader communication skills [6, 7] and as such it may be desirable to assess it [8] for a number of reasons. Empathy allows clinicians to better understand patient perspectives, including the worries or concerns of patients, which may help in knowing where to direct emotional support and information. It is likely to be important for good team-working, good bedside manner and the ability to develop helpful therapeutic alliances, leading to better patient care [9, 10]. It enables the student or doctor to ask appropriate questions at appropriate times, and gauge what is required for a successful interaction [11]. Genuine empathy may also lead to the student or doctor being curious about the patient experience and their story [12], improve patient trust and engagement with suggested treatments, and reduce anxiety for many patients [13, 14]. Medical students’ well-being appears to be better, with lower levels of distress and possibly burnout when they have a good ability to empathize [15, 16].

Whilst many studies report assessing more general communication skills in examinations [17], or using clinician rated scores of communication skills in ward or clinic settings [18], empathy more specifically is less frequently reported as being assessed. Some studies use self-report questionnaires to assess empathy [10, 19, 20]. One study used clinical observer ratings in clinical examinations for 57 medical students [21] with Rating Scales for the Assessment of Empathic Communication in Medical Interviews [22], which comprises 9 items each with a 7 point Likert Scale (6 measuring empathy). Reviews report a variety of ratings of process or outcome in standardized encounters with real or simulated patients [18, 23], few report assessing empathy by those directly in the encounter (e.g. patient or simulated patient). In a review of 13 peer reviewed studies by Stepien & Baernstein [3] that sought to measure empathy in medical students, none used patient perceptions or simulated patients to assess empathy. The History-taking Rating Scale has some items that ask the observer about student patient interaction (for example about ‘the student’s expressed understanding of what the patient is feeling and communicating’), but less than a third of these items are empathy related and they are not validated separately from the whole scale [24].

Actor Simulated Patients (SPs) have been used in previous studies to rate history taking, physical examination skills and general communication skills [25, 26], but few studies report SP assessment of empathy specifically in examinations. One study of US students at the end of their third year of medical training in one school did so using a five item rating scale and a single global scale to rate 10 OSCE stations in which SPs encountered the students [27]. This was alongside the self-reported Jefferson Scale of Physician Empathy, which was developed to measure ‘cognitively defined empathy’ (as distinct from affective or emotional empathy). They found that SPs rated Asian Americans lower than white Americans even though there were no differences on the self-reported empathy questionnaire between these groups. Another study in Korea found very mixed results when correlating emotional empathy ratings with academic performance in clinical examinations [28].

Our study sought to bring empathy ratings into clinical short case final examinations and test their usefulness using a simple general construct of empathy (including both cognitive and affective aspects) compared to other final year clinical examination scores. We also set out to see whether SP actor ratings were comparable to clinical examiner ratings, and how they correlated to independent examinations scores. We hypothesised that SPs would be good at rating empathy in the clinical examination encounter and that empathy ratings would be able to predict candidates with low overall examinations scores.

## Methods

Ethics approval to use the empathy scores from examiners and simulated patients as a formative score only in the final year examination was granted by the Hull York Medical School Ethics Committee. Participants were 133 students at Hull York Medical School (England/UK) sitting their final examinations in May 2012. The final year examination consists of 6 OSCE (Objective Structured Clinical Examination) stations each of 7 minutes in length and four 30–minute OSLER (Objective Structured Long Examination Record) stations, which are cases with real patients. Scores for each of these ten stations are combined for an overall clinical examination score [29]. This is a sequential examination in which those students with low scores, from this first day who do not demonstrate satisfactory competence are called back for further clinical examinations the next day.

In the six OSCE stations, two examined procedural skills of cannulation and completing a prescribing chart and four OSCE stations explicitly examined interactional areas around communication, teamwork, and patient interactions. These were:

1. explaining diagnosis of cancer,
2. responding to concerns about the conduct and performance of a colleague,
3. discharge planning and
4. suicide risk assessment.

OSCE stations were scored on a six point scale (A, B, C+, C-, D, E) with A (excellent) to E (unsatisfactory). The descriptors for each grade are shown in Table 1.

**Table 1:** **OSCE and OSLER grade descriptors**

A	Capable in all components to a high standard
B	Capable in all components to a satisfactory standard and high standard in many
C+	Capable in all components to a satisfactory standard
C-	Capable in a majority of components to a satisfactory standard, inadequacies in some components
D	Capable in a minority of components. No serious defects
E	Capable in a minority of components. One or more serious defects

For these four interactional OSCE stations, as part of our research, we additionally asked the single clinical examiner and the SP to independently rate empathy on a 5 point scale (with 5 being the highest rating and 1 the lowest).

Two psychiatrists and two general practitioners (all clinical tutors) formulated the empathy descriptors for the 5 point rating (see Table 2). These were derived from two strands of the research literature. The first relates to cognitive aspects of empathy where the students can recognise that the patient has their own thoughts, intentions or beliefs [30]. The second relates to the emotional component where students recognise the emotions of the patient and demonstrate an ability to tune in to them and adapt their responses accordingly [31]. The precise wording was chosen pragmatically to be appropriate for the clinical examination context. Clinical examiners and SPs were given instructions as part of the training before the exams on using the guide descriptors shown in Table 1, but also were asked to consider the term “empathy” in the rating to relate to “an ability to understand and respond to the thoughts, feelings and sensations of the other person”; in other words, a broad concept of empathy incorporating both cognitive and affective empathy.

**Table 2:** **Empathy grade descriptors**

5	Excellent Empathy skills. The candidate tunes consistently well to the patients perspectives, knowledge and concerns and develops a good rapport.
4	Good empathy skills. The candidate develops good rapport, but does not always respond to the patient’s questions or concerns or explain things in appropriate emotional tone or language.
3	Some empathy skills in evidence, the candidate appears to understand the patient’s perspective at some points but less at other points.
2	Some empathy in evidence at times, but largely misses what the patient’s needs are, and their concerns, regularly uses inappropriate emotional tone or language.
1	Poor empathy. There is little attempt to understand the patients needs, factual information is delivered without sensitivity. Consistently uses inappropriate emotional tone or language.

A maximum empathy score for a student would be 40 with 2 raters in each of four stations (range 8–40).

The OSLERs (4 longer clinical cases) were examined by two experienced tutors, one hospital consultant and one general practitioner, rating the students in five areas: gathering information, physical examination, problem solving, patient management, and relationship with patient. The descriptors were the same as for the OSCE and are shown in Table 1.

These OSLERS did not have empathy ratings as a separate judgement. We wished to see if the empathy scores correlated with existing measures of patient interactions and did not want to potentially redirect the examiners too much to consideration of empathy rather than on the existing five criteria with which they were familiar. Scores in these separate exams (OSCEs and OSLERs) were compared to empathy scores in the OSCE stations.

The existing clinical examination uses a scoring system for both the OSCEs and OSLERs, where an A, B or C + is considered as reflecting adequate competence at this stage of their career. Scores of C-, D or E reflect increasingly poor clinical performance and attract ‘penalty points’ of -1 -2 and -3 respectively. The two OSLER examiners’ penalty point scores × 4 patients in 5 areas were added together along with the six penalty point scores from the OSCE stations with one examiner per station. The SPs did not rate students on clinical performance.

The total (OSCE + OSLER) penalty point scores could therefore potentially range from 0 to 138. The actual range of penalty points was 0 to 41. All students see the initial 6 OSCE stations and 4 OSLER patients. However, the full sequential examination requires students who have not demonstrated competence over these initial 10 stations, based on accumulating penalty points from C-, D and E ratings, to return for a second part of the examination to gather more data on which to base a decision. The cut-off point is set at a predetermined level to bring back approximately a third of students with the lowest scores. After the second part of the examination, consisting of another 6 OSCE stations and 4 OSLER patients, an overall passmark is set by the Borderline Groups method. The details of the exact procedures can be found in Cookson et al. [29].

## Results

Empathy scores on each OSCE station were given by the clinical tutor and the simulated patient. The mean empathy score per station given by SPs was 3.64 (SD = .91) and by clinical tutors was 3.69 (SD = .81), with both groups using the full range of possible scores (1–5). The range of total scores given across all students was 20–38 with a possible range of 8–40. The reliability of the Empathy scale scores measured by Cronbach’s alpha was .74.

**Figure 1:**
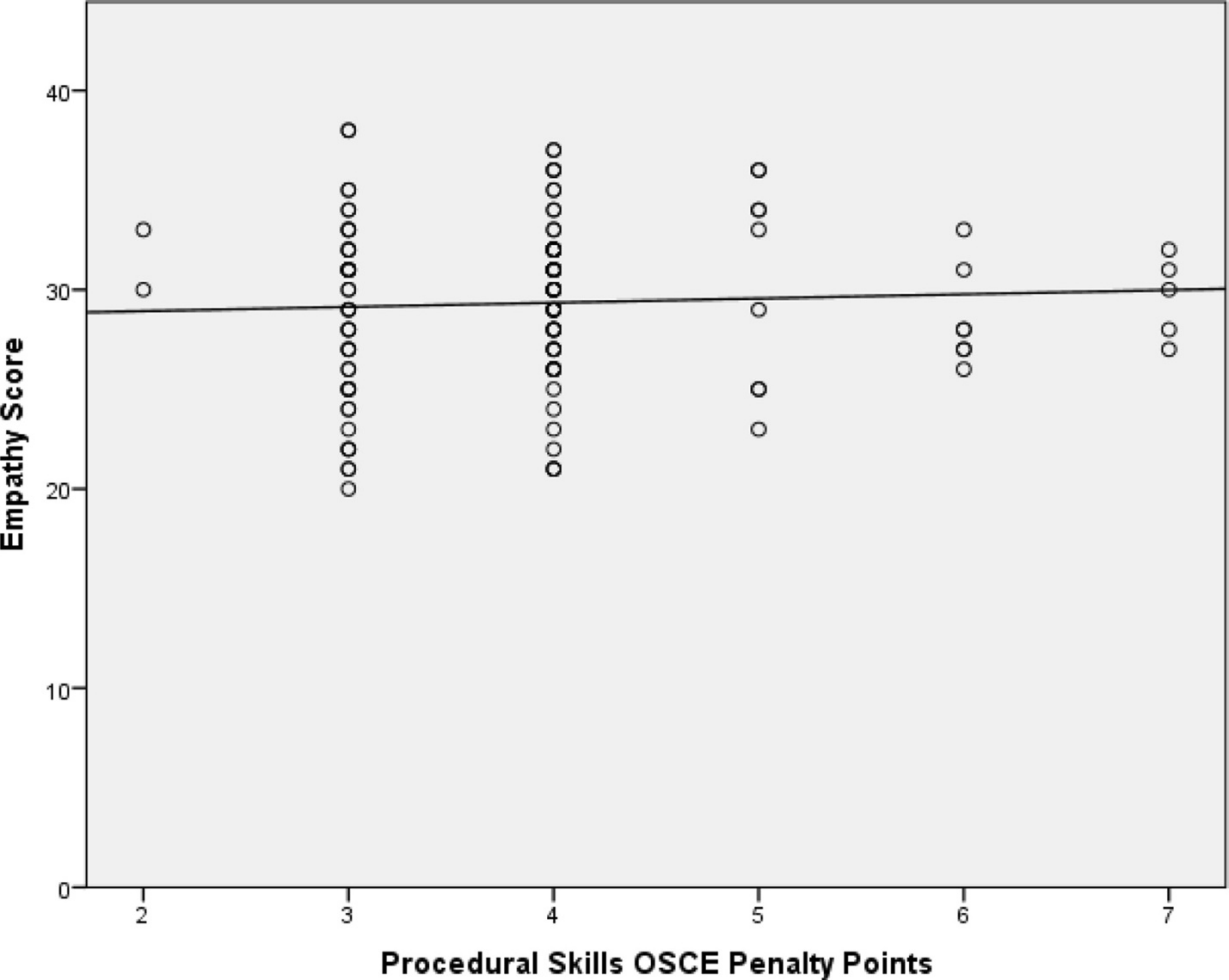
**Empathy score versus penalty points on procedural OSCE stations.** Empathy score total awarded by examiners and simulated patients versus penalty points accumulated on the two procedural OSCE stations (Spearman’s correlation r_s_ = 0.07, p = 0.43).

Spearman’s correlations were run for the empathy scores and various of the examination scores. There was no correlation between empathy scores and penalty points on the two procedural skill based OSCE station scores (r_s_ = .07, see Figure 1).

**Figure 2:**
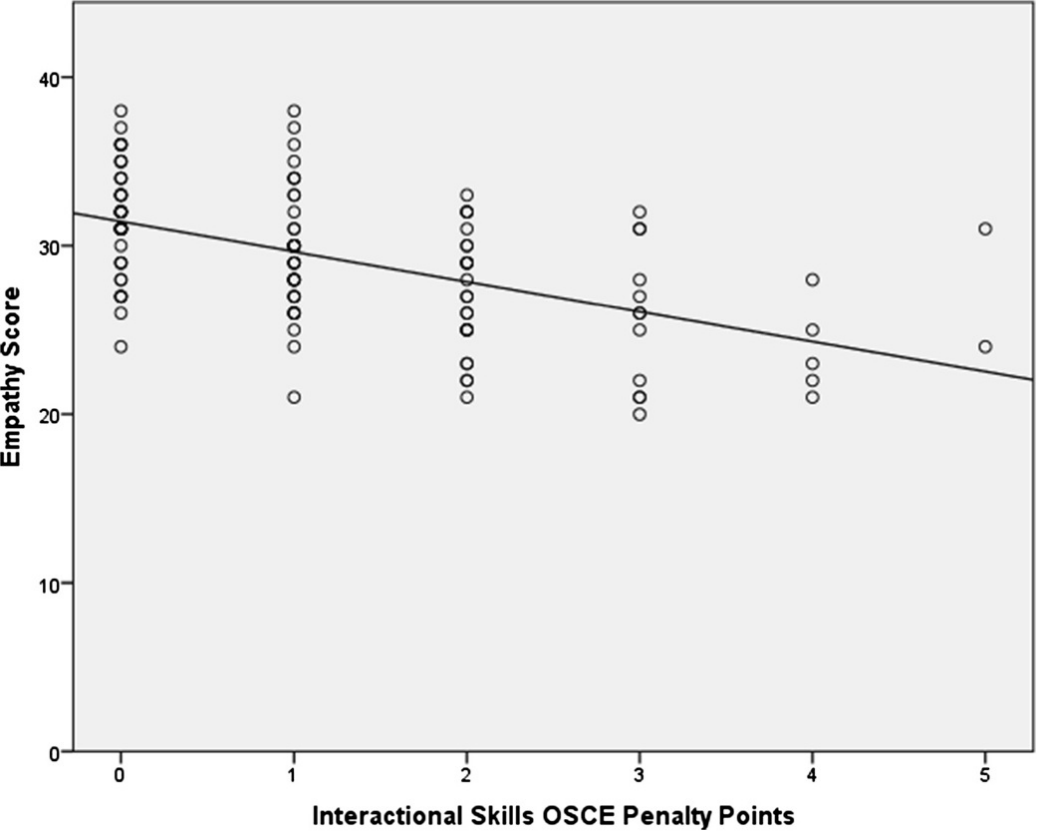
**Empathy score versus penalty points on interaction based OSCE stations.** Empathy score total awarded by examiners and simulated patients versus penalty points on the four interaction based OSCE stations (Spearman’s correlation r_s_ = −0.54, p < 0.0001).

However, there was a significant negative correlation of r_s_ = −.54 (n = 133; p < 0.0001) between empathy scores and penalty points for the four OSCE stations involving interactional and communication skills (Figure 2).

**Figure 3:**
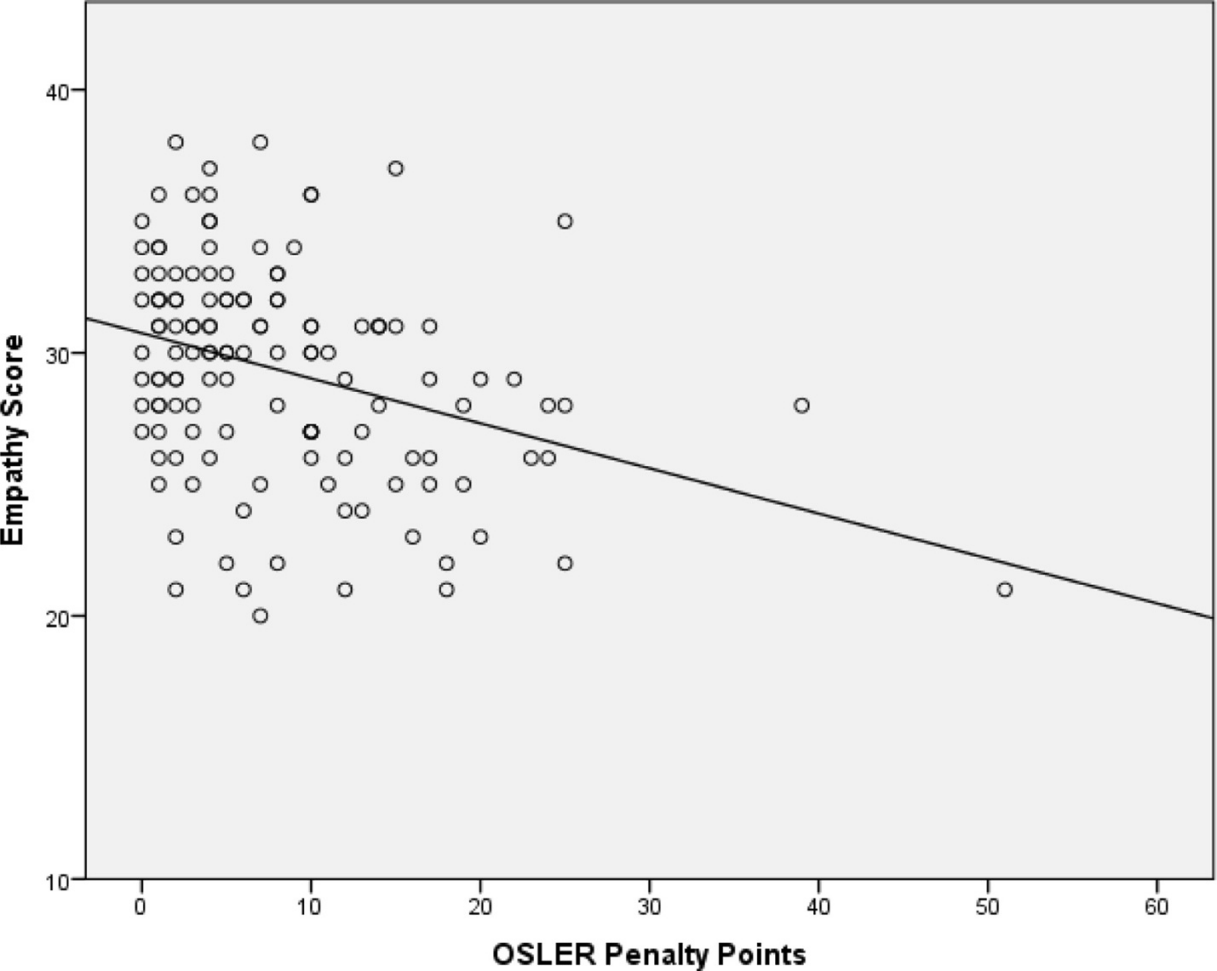
**Empathy score versus total OSLER penalty point score.** Empathy score total awarded by examiners and simulated patients versus total OSLER penalty point score (Spearman’s correlation r_s_ = −0.38, p = <0.001).

Using the penalty point scoring on the OSLERs, the correlation between the empathy scores accumulated on the OSCE stations and the OSLER penalty point scores was r_s_ = −.38 (n = 133; p < .0001). The data are shown in Figure 3. One student accumulated 59 penalty points. When this outlier is removed from the analysis, the correlation is r_s_ = −.29 (n = 132; p < .001).

Thirty nine students were called to the second part of the examination. The average empathy score for the 94 students not returning was 30.2 and the average for the 39 sitting the second part of the examination was 27.2. An independent t-test for the two groups indicates a significant difference (t = −4.2, df = 131, p < .0001). The Cohen’s d = 0.81, indicating that the two groups’ means differ by 0.81 standard deviation, a ‘large’ effect size [32].

### Inter-rater reliability

Inter-rater reliability was measured between clinical examiners and actor simulated patients in the OSCE, using the individual empathy scores for each station. The intra-class correlation (ICC) is a measure of inter-rater reliability used when students are being rated by a number of different raters and yields a value between 0 to 1. The ICC (1,1) was used, which is a one-way random single measure ICC. This was chosen because each subject was assessed by a different set of randomly selected raters. Overall the reliability as measured by the ICC is 0.645 (95% CI 0.593-0.692) indicating substantial agreement [33]. The station *Addressing concerns about a colleague’s conduct* shows excellent reliability of 0.754 (0.670-0.819), with the lowest reliability on the station *Suicide risk assessment* 0.502 (0.363-0.619). Other ICCs were *Explaining a cancer diagnosis* 0.603 (0.481-0.701) and *Discharge planning concerns* 0.658 (0.549-0.745).

## Discussion

In relation to the first question posed about how independently assessed empathy scores in clinical finals examinations relate to performance in the clinical examinations more generally, we found that empathy scores show significant correlation with the interaction based OSCE stations and virtually no correlation with the skills based stations. This could be because a good ability to empathize is more likely to influence performance in the interaction based OSCEs than the practical OSCEs where neither involved interaction with a patient or colleague. Students with poor empathy scores were distributed across the range of practical skill scores suggesting that empathy is not discriminatory in these stations. By contrast, in the clinically based OSCEs those students with poor empathy scores were more likely to do worse on the stations, accumulating more penalty points.

The empathy scores given in the OSCE stations showed a significant correlation with the OSLER penalty point scores given by a completely different set of raters, r_s_ = −.38 or r_s_ = −.29 with outlier removed. Those with low empathy performed worse on these long case clinical examinations. It may be that though examiners are not explicitly being asked to measure empathy when rating OSLER performance in areas such as ‘gathering information’ and ‘patient relationship’, the ratings may reflect allied or overlapping qualities. It is relevant that this correlation is moderately high given that the OSLER examinations and OSCE empathy raters are completely separate from each other. This shows that a skill measured in one context (OSCE) appears to be relevant in a clinical examination in a different independent context (OSLER clinical examinations), giving some indication of construct validity for the empathy measure.

This is also important because it has been argued that simulated patients, or indeed, any examination context, cannot validly assess empathy because the situation is too artificial [34]. While it is true that a simulated patient encounter is not “real”, this study has shown that empathy ratings by SPs and by clinicians correlate significantly, though not perfectly, with ratings of communication and relationship with real patients in the OSLER. This would indicate some veracity for the claim that these ratings are capturing some aspects of a student’s ability to relate to a patient, particularly when these ratings did not correlate with performance on procedural skills stations, which would be the case if a high empathy rating was simply a proxy for a generally good student. There is also the larger question of how a student’s ability can be assessed before they begin practicing independently. Is observing a student with a real patient in a GP clinic authentic enough, or is it too artificial because the student is aware of the gaze of surveillance? These are questions that should be seriously explored, but the judgement of simulated patients should not be dismissed as irrelevant, even if the “performances” of SP and student are not the actual target encounter.

It is reasonable to ask whether an individual’s ability to empathize could be better measured using a standardised questionnaire. Empathy ratings using standard validated questionnaire scales in medical school do seem to have some predictive validity when compared to subsequent ratings by clinical tutor/training programme directors post-qualification [35] and so research in this area is worthy of more attention. However, fixed self-rating questionnaires such as these are not as useful in assessment for medical school courses, as students are adept at learning to answer questions of accessible fixed questionnaires in a manner to ensure progression [36]. Furthermore results may not always correlate well with demonstrated empathy [37], which may vary with each encounter. We chose not to use a standardized measure alongside our ratings for this reason. It has also been suggested that paper tests cannot capture behavioural aspects of empathy [24] and empathy is a prime area where students ‘may not know what they don’t know’. Answering questions about empathy at an intellectual level may be very different from demonstrating it in clinical examinations or clinical practice. The results of this study suggest that it would be useful to conduct more research on the utility, reliability and validity of assessing empathy in clinical examinations.

In relation to the second question about whether an SP (actor) can rate empathy and how this compares to the clinical tutor rating, we found that inter-rater correlation was reasonably good. Whilst it is known that empathy ratings from questionnaire assessment correlate well with broader communication skill ratings by simulated patients [19], few studies have used simulated patients as a mechanism for assessing empathy [3], despite the fact that SPs are directly in the clinical encounter and therefore likely to be in a position to assess empathy. There may be a concern that actors may not be skilled enough to rate students but we found good inter-rater reliabilities with empathy ratings of clinical examiners who were assessing, even though they were rating independently from each other. All of the simulated patients used in this examination were very experienced and supported medical student teaching throughout all five years of the medical school. They were well versed in the expected level of competence of the fifth year students and given annual training for their role.

## Conclusions

This study raises the question of how such measures of empathy might be used. Given their apparent reliability and the fact that perspective taking and empathy are often explicitly stated in medical school learning outcomes, it seems reasonable to use feedback in a formative way. Simulated patients’ feedback can easily be included in communication skills laboratory sessions, and communication skill workshops. They can be used in video feedback sessions or in certain types of problem based learning sessions. The correlation with overall examination performance in our study gives encouragement to further research on whether these measures (or measures like them) can be used across different medical schools, and to develop more evidence of their use for summative assessments.

There are a number of limitations with this study. The first is that these clinical examinations are snapshots in a contrived setting and it begs the question whether empathy can really be adequately assessed in these circumstances. The second is that empathy is a broad concept with different definitions, and choosing a clear construct to assess is not a straightforward task. Clear guidance also needs to be given to assessors since they may conceptualise empathy in different ways leading to them assessing for different things. However, the empathy scores from the OSCE stations did show good correlation with the scores from long patient encounters in the OSLER, which were rated by a different set of examiners, indicating that this may be a reliable assessment of demonstrable behaviours in patient interactions. This study was carried out in one medical school and further replication work would be advisable. Finally, assessors may have their own attributions based on potentially unrelated factors, such as ethnicity, that influence the way they score the students on empathy [27], and there needs to be adequate training and screening of raters to ensure no discrimination takes place. Nevertheless, the correlations found here with other areas of performance are worthy of note. Further work needs to be done to explore whether scores relate to well validated questionnaire-based empathy constructs, and are predictive of clinical performance in the real world.

What we have found is that empathy scores on OSCE stations are meaningfully related to ratings on longer patient cases (OSLERs) and overall clinical examinations of patient encounters. We also found that simulated patients can effectively rate empathy and give valuable insights into the clinical interaction that correlate moderately well with the observing clinical examiners.
